# F214. PSYCHOLOGICAL INTERVENTIONS FOR POSITIVE SYMPTOMS IN SCHIZOPHRENIA: A NETWORK META-ANALYSIS

**DOI:** 10.1093/schbul/sby017.745

**Published:** 2018-04-01

**Authors:** Irene Bighelli, Stefan Leucht

**Affiliations:** Technische Universität München

## Abstract

**Background:**

There is rising awareness about the need of multi-disciplinary approaches integrating psychological treatments for schizophrenia, but a comprehensive evidence base on their relative efficacy is lacking. Conventional pairwise meta-analyses cannot provide a hierarchy based on the randomised evidence. We aimed to integrate the available evidence to create hierarchies of the comparative efficacy, acceptability and tolerability of psychological interventions for schizophrenia.

**Methods:**

We performed a network meta-analysis (which uses both direct and indirect comparisons) of randomized controlled trials on psychological treatments aimed at positive symptoms in the acute treatment of schizophrenia, compared with another psychological intervention or with a no treatment condition (waiting list, treatment as usual). We excluded trials done in patients with predominant negative symptoms, concomitant psychiatric disorders or medical illnesses, and those done in first episode or stable patients. Published and unpublished studies were sought through database searches, trial registries and websites. Study selection and data extraction were conducted by at least two independent reviewers. Our primary outcome is the change in positive symptoms on a validated rating scale. Secondary outcomes include number of dropouts, overall and negative symptoms of schizophrenia, response, relapse, adherence, depression, quality of life, functioning and adverse events. Analyses were conducted in R within a frequentist framework. The risk of bias in studies has been evaluated using the Cochrane Risk of Bias tool and the credibility of the evidence will be evaluated using an adaptation of the GRADE framework to NMA, recommended by the Cochrane guidance. Subgroup and sensitivity analyses will be conducted to assess the robustness of the findings. The protocol of this review has been registered in Prospero (registration number: CRD42017067795).

**Results:**

After screening 20196 references for title and abstract and 2555 full text articles, we identified 58 suitable trials, for a total of 3956 participants.

Regarding primary outcome positive symptoms, only Cognitive Behavioural Therapy was significantly more effective than treatment as usual, with a standardised mean difference of -0.59 [95% credible interval -1.03; -0.16].

The standardised mean differences with 95% credible intervals for other interventions were: Acceptance and Commitment Therapy -0.07 [-2.12; 1.98], Cognitive Therapy -0.18 [-1.92; 1.55], Hallucination Treatment -0.69 [-2.40; 1.01], Metacognitive Therapy -0.26 [-1.16; 0.64], Mindfulness -0.26 [-2.14; 1.62], with heterogeneity tau^2^ = 0.6942.

Data analyses on other outcomes are ongoing.

**Discussion:**

We are going to investigate the possible sources of heterogeneity with the pre-planned subgroup analyses: number of sessions, study duration, individual versus group setting, expertise of the therapist and baseline severity.

A network meta-analysis is the only methodology that allows the production of hierarchies of interventions for treatment of schizophrenia. Such hierarchies, saying which treatment is likely to be the best, the second best and so on, are essential for guideline development. The results of this study are therefore likely to provide knowledge of great impact for treatment decisions.

This project has received funding from the European Union’s Horizon 2020 research and innovation programme under the Marie Skłodowska-Curie grant agreement No 701717.

